# Coincidence detection of RAB11A and PI(3)P by WIPI2 directs autophagosome formation

**DOI:** 10.18632/oncotarget.26829

**Published:** 2019-04-05

**Authors:** Mariella Vicinanza, Claudia Puri, David C. Rubinsztein

**Affiliations:** David C. Rubinsztein: Department of Medical Genetics, Cambridge Institute for Medical Research, Cambridge, UK; UK Dementia Research Institute, The Keith Peters Building, Cambridge Biomedical Campus, Cambridge, UK

**Keywords:** autophagy, RAB11, WIPI2, PI(3)P, coincident detection

Macroautophagy, hereafter referred to as autophagy, is a process that delivers cytoplasmic material to lysosomes for degradation. When nutrients are scarce, autophagy sustains cellular renovation by recycling cellular constituents (amino acids and fatty acids) for anabolic processes. In nutrient-replete conditions, autophagy maintains cellular homeostasis by selectively degrading disease-related ‘cargoes’ (pathogenic aggregate-prone proteins, damaged organelles, invasive bacteria).

The first morphologically recognizable precursor of the autophagic process is a sac-like structure (phagophore) that first expands as a flat double-membrane sheet and then bends and closes into a spherical double-membrane organelle (autophagosome). Nucleation, elongation and closure are governed by the conserved ATG (AuTophaGy-related) proteins, many of which are recruited at the autophagosome formation site [1]. During autophagosome formation, the ATG8 ubiquitin-like proteins (LC3/GABARAP) are conjugated to the lipid phosphatidylethanolamine (PE) and associate tightly with autophagosomal membranes. The site of LC3 conjugation is specified by the ATG16L1 complex, which is recruited by WIPI2, a member of the ATG18/WIPIs (WD-repeat protein Interacting with PhosphoInositides) family [2].

Despite being defined as the membrane to which LC3 becomes conjugated, the identity of the “isolation membrane” on which autophagosomes form is still enigmatic [3]. In order to address this key question, we further investigated the molecular underpinnings of WIPI2/ATG16L1 membrane recruitment. Conventionally, a local increase in phosphatidylinositol 3-phosphate (PI(3)P) formed by the class III phosphatidylinositol 3-kinase (VPS34/Beclin1 complex) at the “isolation membrane” is considered the docking site for WIPI2. However, protein-phosphoinositide interactions generally have low affinities (in the micromolar range) and thus may be insufficient to enable efficient protein recruitment at physiological concentrations [4, 5]. In addition, PI(3)P is found in multiple sites in the cell (i.e. early/late endosomes) where WIPI2/ATG16L1 are not detected [6, 7] (Figure 1A). Hence, we speculated that PI(3)P may not act alone to recruit WIPI2/ATG16L1 to nascent phagophores and that an additional protein-protein interaction may be required to increase the targeting efficiency and the binding strength of WIPI2/ATG16L1 to membranes.

**Figure 1:**
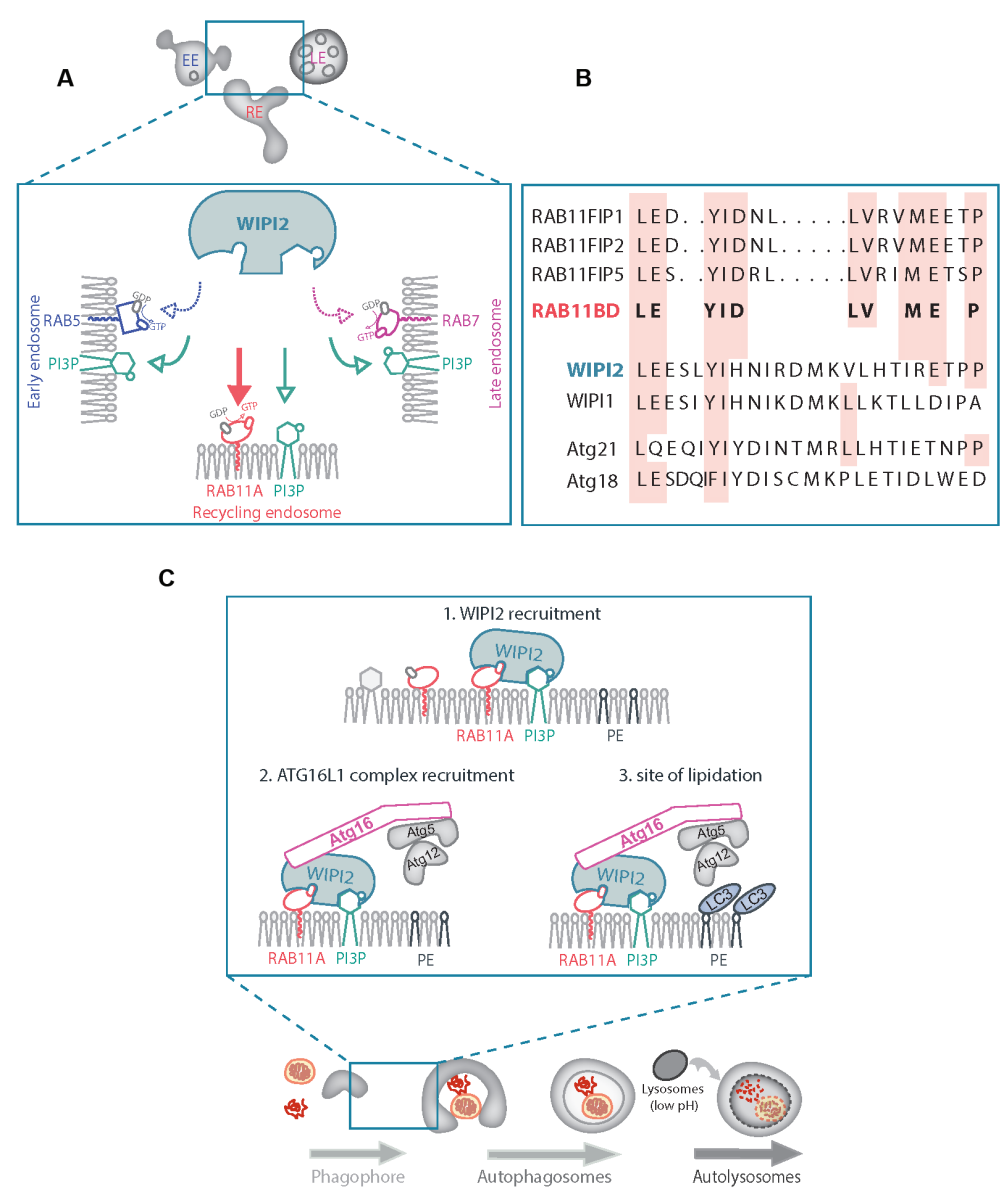
Coincident detection of PI(3)P and RAB11A by WIPI2 during autophagosome formation **A.** PI(3)P cooperates with RAB11A, a core component of recycling endosomes, to stabilize WIPI2 on membranes. Although multiple endosomal compartments are enriched in PI(3)P (i.e. early/late endosomes), WIPI2 binds recycling endosomes with higher specificity because it also binds RAB11A in concert with PI(3)P. **B.** Alignment of amino acid sequence of the consensus RAB11-binding domain (R11BD) of RAB11FIPs, known RAB11 effectors, with human and yeast counterparts of WIPI2 and WIPI1. **C.** Coincident detection of PI(3)P and RAB11A by WIPI2 (1) and recruitment of ATG16L1 complex (2) occur on the RAB11A-positive membranes that become the membrane to which LC3 becomes conjugated (3) and the platform where autophagosome assembly occurs.

Using mass spectrometry, biochemical and microscopy approaches, we recently identified the small GTPase RAB11A as a protein that acts in concert with PI(3)P to target the WIPI2/ATG16L1 complex to the “isolation membrane” [6]. WIPI2 protein sequence analysis unveiled the presence of a RAB11-binding signature (RBD), conserved among RAB11 effectors (Figure 1B). Mutations of these residues (or silencing of RAB11A) resulted in WIPI2 disappearance from nascent autophagosomes, defective autophagosome assembly (lack of ATG16L1 and LC3 on forming autophagosomes) and inefficient autophagic cargo (autophagy adaptor p62, mutant huntingtin exon 1 aggregate and depolarized mitochondria) degradation. The binding sites for RAB11A and PI(3)P are distinct (Figure 1A) and *in vitro* the combination of RAB11A and PI(3)P were additive for WIPI2 binding to liposomes. In living cells, a combination of RAB11A and PI(3)P is a strict requirement for having WIPI2 correctly placed at forming phagophores. Most PI(3)P and PI(3)P effectors concentrate on RAB5-positive early endosomes. By contrast, WIPI2 does not associate with this compartment (Figure 1A). However, if we artificially localized RAB11A to early endosomes, then we could relocate WIPI2 to these structures, highlighting the specificity of WIPI2-RAB11A interaction.

RAB11A is a core component of recycling endosomes, and we found that WIPI2 also colocalised with other markers for this compartment like RAB10 and transferrin receptor. In line with the “coincidence detection” model where phosphatidylinositides act in concert with colocalized GTPases to modulate the recruitment of shared effectors [4], our data suggest that PI(3)P and active RAB11A on recycling endosomes generate a transient platform enabling WIPI2/ATG16L1 complex recruitment, LC3/GABARAP conjugation and autophagosome assembly (Figure 1C). Indeed, by confocal immunofluorescence microscopy studies in autophagy-induced cells, we could further show that RAB11A-positive membranes accumulated core ATGs (PI(3)P-related proteins, like DFCP1 and ATG14/Beclin 1; ATG8 ubiquitin-like proteins, like LC3/GABARAP; and closure components like ATG2A) and engulfed autophagic cargoes (damaged mitochondria and mutant huntingtin aggregates).

This enabled characterisation of the RAB11A-positive membranes (likely to be the recycling endosomes) as a platform on which key ATG proteins dock to assist the evolution of RAB11A subdomains into phagophores. While our findings challenge the conventional view of autophagosome biogenesis occurring on the ER (endoplasmic reticulum), our data are consistent with studies showing a role for the ER in autophagosome biogenesis by contributing some components of the LC3/GABARAP lipidation machinery (like ATG3) [8].

## References

[1] Galluzzi L (2017). EMBO J.

[2] Dooley HC (2014). Mol Cell.

[3] Lamb CA (2013). Nat Rev Mol Cell Biol.

[4] Carlton JG (2005). Trends Cell Biol.

[5] Di Paolo G (2006). Nature.

[6] Puri C (2018). Dev Cell.

[7] Puri C (2013). Cell.

[8] Ge L (2013). Elife.

